# The Aging Brain With HIV Infection: Effects of Alcoholism or Hepatitis C Comorbidity

**DOI:** 10.3389/fnagi.2018.00056

**Published:** 2018-03-22

**Authors:** Natalie M. Zahr

**Affiliations:** ^1^Neuroscience Program, SRI International, Menlo Park, CA, United States; ^2^Department of Psychiatry and Behavioral Sciences, Stanford University School of Medicine, Stanford University, Stanford, CA, United States

**Keywords:** alcohol use disorder, alcoholism, hepatitis C, magnetic resonance imaging, magnetic resonance spectroscopy, diffusion tensor imaging, neuropsychological tests

## Abstract

As successfully treated individuals with Human Immunodeficiency Virus (HIV)-infected age, cognitive and health challenges of normal aging ensue, burdened by HIV, treatment side effects, and high prevalence comorbidities, notably, Alcohol Use Disorders (AUD) and Hepatitis C virus (HCV) infection. In 2013, people over 55 years old accounted for 26% of the estimated number of people living with HIV (~1.2 million). The aging brain is increasingly vulnerable to endogenous and exogenous insult which, coupled with HIV infection and comorbid risk factors, can lead to additive or synergistic effects on cognitive and motor function. This paper reviews the literature on neuropsychological and *in vivo* Magnetic Resonance Imaging (MRI) evaluation of the aging HIV brain, while also considering the effects of comorbidity for AUD and HCV.

## Introduction

The concept and benefits of combining multiple drugs for treatment of Human Immunodeficiency Virus (HIV) infection was introduced in 1996 (Gulick et al., 1997; Hammer et al., 1997). Polydrug therapies, referred to as highly active Antiretroviral Therapy (HAART) or equivalently, combination Antiretroviral Therapy (cART) were quickly incorporated into clinical practice, resulting in significantly reduced rates of hospitalizations, Acquired Immune Deficiency Syndrome (AIDS), and death (Moore and Chaisson, 1999). Because highly effective, combination regimens have since been the default in ART, and because newer one-pill options make use of the word “combination” obsolete, there has been a recent trend in referring to HIV treatments as ART instead of HAART or cART (Myhre and Sifris, 2017). Despite the effectiveness of ART in reducing HIV viral load and improving immune function, HIV infection continues to have major untoward public health and clinical consequences (Powderly, 2002).

Each year in the United States (US), 55,000–60,000 new infections are reported, with an estimated total of ~1.2 million infected individuals. In 2013, people ≥50 years old accounted for 17–26% (or up to 312,000 individuals) of the HIV population (Center for Disease Control and Prevention, 2013). Older individuals are more likely to be diagnosed later in the course of the disease; indeed, 40% of people ≥55 are diagnosed with AIDS at the time of HIV diagnosis (Lindau et al., 2007; Brooks et al., 2012; Center for Disease Control and Prevention, 2015, 2016a,b). As individuals infected with HIV live longer (e.g., Thompson and Jahanshad, 2015), they are likely to accrue central nervous system (CNS) risk from factors such as substance use disorders (e.g., alcoholism), comorbid infections [e.g., hepatitis C virurs (HCV)], and medical conditions associated with ART treatment (Woods et al., 2004).

The considerable comorbidity of HIV infection and alcoholism (Cook et al., 2001; Miguez et al., 2003; Samet et al., 2004, 2007; Conigliaro et al., 2006; Fuller et al., 2009; Bonacini, 2011) poses a greater public health burden than either condition alone. Individuals who drink heavily or have been diagnosed with DSM-IV alcohol abuse/dependence or DSM5 alcohol use disorder (AUD) are more likely to engage in risky sexual behaviors, delay testing for HIV, and postpone treatment (Fritz et al., 2010; Howe et al., 2011). Conversely, AUD may make it difficult for infected patients to follow the complex medication regimen prescribed to treat HIV or interfere with basic mechanisms of pharmacological treatment. HCV infects ~25% of HIV-infected people in the US (Center for Disease Control and Prevention, 2011). HIV patients co-infected with HCV, who are also likely to drink heavily (>50 g alcohol/day), have higher mortality rates than low or moderate drinkers (Bonacini, 2011).

Cross-sectional studies have been instrumental in identifying brain regions and systems affected in HIV infection, but are limited to speculation about the potential interaction of these effects with aging and variables that change with disease progression or mitigation (e.g., Ances et al., 2012). Inconsistency in findings may be, at least in part, attributable to the cross-sectional examination of a dynamic disease. Indeed, any conclusion determining whether aging interacts and exacerbates the untoward effects of HIV infection, or alternatively, whether disease progression is a greater contributor than age to decline requires longitudinal study of the relevant variables in HIV-infected groups (e.g., Holt et al., 2012; Spudich and Ances, 2012).

**Figure 1:**
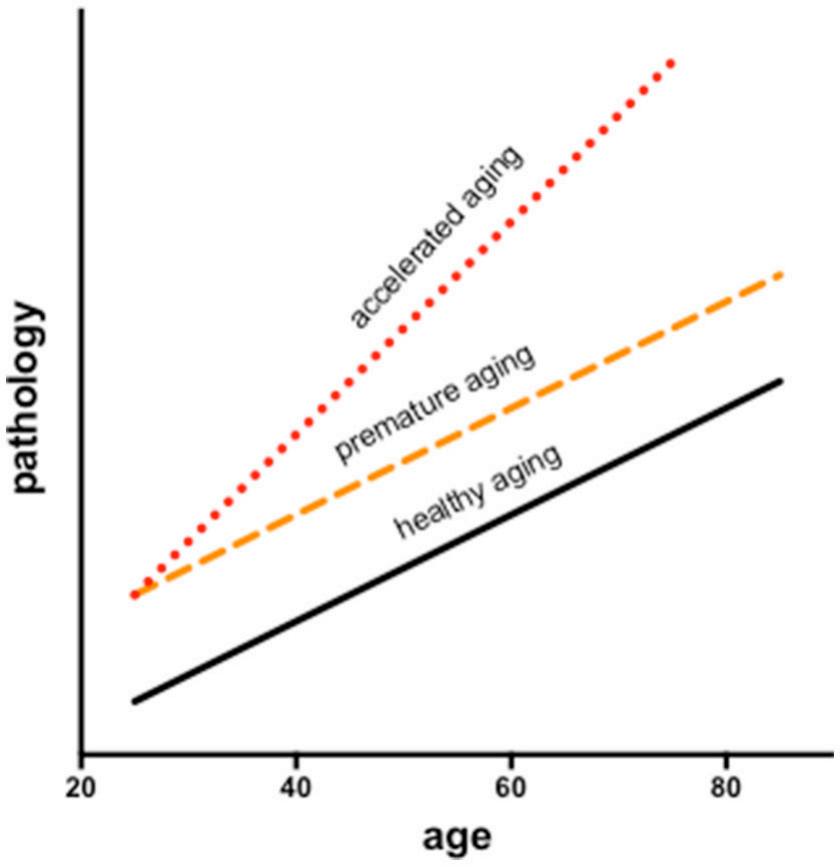
Longitudinal modeling of the interactions of aging and HIV consider two potential trajectories: premature and accelerated aging. Infection may facilitate processes associated with aging resulting in premature aging, during which changes occur earlier but in parallel to normal aging or accelerated aging, wherein changes occur at a faster rate than in normal aging.

In longitudinal modeling of the interactions of aging and HIV, two potential trajectories are often considered: premature (additive) or accelerated (synergistic) aging. Infection may facilitate processes compromised by older age resulting in premature aging, during which changes occur earlier but in parallel to normal aging or accelerated aging, wherein changes occur at a faster rate than in normal aging (Figure 1). Results may also depend on the metric evaluated (e.g., neuropsychological performance vs. brain volumes).

In the following, the literature on brain structure and function in HIV and relevant comorbidities (i.e., AUD, HCV) is reviewed, with a focus on longitudinal studies to help clarify the independent or interactive effects of older age. Table 1 provides a list of references used herein, concentrating on manuscripts published after 2007, for HIV and each comorbid condition, also indicating cross-sectional or longitudinal studies. Table 2 summarizes key findings highlighted in this review.

**Table 1:** List of references used in this manuscript focused on publications after 2007 and listed in alphabetical order.

	**Neuropsychological testing**			**Structural MRI**		
	**AUD**	**HIV**	**HCV**	**AUD**	**HIV**	**HCV**
Cross sectional	Chopra and Tiwari, 2012; Noble and Weimer, 2014; Oscar-Berman et al., 2014; Vassar and Rose, 2014; Wilcox et al., 2014; Le Berre et al., 2017	Antinori et al., 2007; Robertson et al., 2007, 2009, 2011; Dawes et al., 2008; Evans et al., 2008; Foley et al., 2008, 2013; Hardy and Vance, 2009; Ciccarelli et al., 2011; Morgan et al., 2011; Sullivan E. V. et al., 2011; Kranick and Nath, 2012; Bonnet et al., 2013; Gabbai et al., 2013; Nakazato et al., 2014; Arentoft et al., 2015; Becker et al., 2015; Jacks et al., 2015; Sheppard et al., 2015; Vassallo et al., 2015; Ma et al., 2016; Prakash et al., 2016; Sacktor et al., 2016; Adoukonou et al., 2017; Benevides et al., 2017; Gomez et al., 2017; Hobkirk et al., 2017; Saylor et al., 2017; Underwood et al., 2017b	Karaivazoglou et al., 2007; Thein H. H. et al., 2007; Vigil et al., 2008; Cattie et al., 2014; Adinolfi et al., 2015; Mathew et al., 2016; Iriana et al., 2017	Chanraud et al., 2007, 2009a; Boutte et al., 2012; Pitel et al., 2012	Dewey et al., 2010; Jernigan et al., 2011; Ragin et al., 2011, 2012; Sullivan E. V. et al., 2011; Tate et al., 2011; Becker et al., 2012; Heaps et al., 2012, 2015; Kallianpur et al., 2012, 2013, 2016; Bernard et al., 2013; Cysique et al., 2013; Fennema-Notestine et al., 2013; Li et al., 2013, 2014; Steinbrink et al., 2013; Haddow et al., 2014; Nishijima et al., 2014; Arentzen et al., 2015; Clark et al., 2015; Janssen et al., 2015; Ortega et al., 2015; Wade et al., 2015; Corrêa et al., 2016a; du Plessis et al., 2016; Hines et al., 2016; Jiang et al., 2016; Narvid et al., 2016; Rubin et al., 2016; Su et al., 2016; Wang et al., 2016; Wendelken et al., 2016; Wright et al., 2016; Castillo et al., 2017; Clifford et al., 2017; Cole et al., 2017; Lake et al., 2017; Sanford et al., 2017; Shin et al., 2017; Underwood et al., 2017a	Weissenborn et al., 2009; Bezerra et al., 2011; Iwasa et al., 2012; Hjerrild et al., 2016
Longitudinal	Fama et al., 2009	Thaler et al., 2015	Kuhn et al., 2017	None identified.	Stout et al., 1998; Cardenas et al., 2009; Ances et al., 2012; Pfefferbaum et al., 2014	None identified.
	**HIV+AUD**			**HIV+AUD**		
Cross sectional	Fama et al., 2007, 2011, 2012, 2016; Rosenbloom et al., 2007; Sassoon et al., 2007, 2012; Míguez-Burbano et al., 2014; McNamara et al., 2017			Cardenas et al., 2007; Durazzo et al., 2007; Rosenbloom et al., 2010; Fama et al., 2011, 2014; Pfefferbaum et al., 2012		
Longitudinal	None identified.			None identified.		
		**HIV+HCV**			**HIV+HCV**	
Cross sectional		Thein H. H. et al., 2007; Hinkin et al., 2008; Martin-Thormeyer and Paul, 2009; Devlin et al., 2012; Sun et al., 2013; Caldwell et al., 2014; Clifford et al., 2015; Martin et al., 2015			Bladowska et al., 2014; Ojaimi et al., 2014; Robinson-Papp et al., 2017	
Longitudinal		Molsberry et al., 2015			None identified.	
	**MRS: neurometabolite imaging**			**DTI: microstructural imaging**		
	**AUD**	**HIV**	**HCV**	**AUD**	**HIV**	**HCV**
Cross sectional	Durazzo et al., 2010; Modi et al., 2011; Hermann et al., 2012	Paul et al., 2008; Chang et al., 2013, 2014; Hua et al., 2013; Harezlak et al., 2014; Vigneswaran et al., 2015; Bairwa et al., 2016	Forton et al., 2008; Bokemeyer et al., 2011; Grover et al., 2012; Bladowska et al., 2013	Chanraud et al., 2009b; Müller-Oehring et al., 2009; Pfefferbaum et al., 2009b; Schulte et al., 2012; Trivedi et al., 2013; Fortier et al., 2014	Stebbins et al., 2007; Chen et al., 2009; Pfefferbaum et al., 2009a; Hoare et al., 2011; Towgood et al., 2011; Du et al., 2012; Jahanshad et al., 2012; Nakamoto et al., 2012; Stubbe-Drger et al., 2012; Leite et al., 2013; Xuan et al., 2013; Zhu et al., 2013; Nir et al., 2014; Corrêa et al., 2015; Wright et al., 2015; Seider et al., 2016; Su et al., 2016; Wendelken et al., 2016; Strain et al., 2017; Tang et al., 2017; Watson et al., 2017	Bladowska et al., 2013; Thames et al., 2015
Longitudinal	None identified.	Lentz et al., 2011; Sailasuta et al., 2012; Gongvatana et al., 2013; Young et al., 2014; Scott et al., 2016; Rahimy et al., 2017	None identified.	None identified.	Chang et al., 2008; Corrêa et al., 2016b	None identified.
	**HIV+AUD**			**HIV+AUD**		
Cross sectional	Zahr et al., 2014			Pfefferbaum et al., 2007		
Longitudinal	None identified.			None identified.		
		**HIV+HCV**			**HIV+HCV**	
Cross sectional		Garvey et al., 2012			Gongvatana et al., 2011; Heaps-Woodruff et al., 2016	
Longitudinal		None identified.			None identified.	

*For EASE of READING, only the 1st author is listed*.

**Table 2:** Summary of findings from manuscripts listed in Table 1.

**Neuropsychological testing**			**Structural MRI**		
**AUD**	**HIV**	**HCV**	**AUD**	**HIV**	**HCV**
	Attention	Attention	Frontal cortex	Frontal cortex	Frontal cortex
**Visuospatial abilities**				Cingulate cortex	
**Emotion regulation**				Motor cortex	
Psychomotor speed	Psychomotor speed	Psychomotor speed		Parietal cortex	
Memory	Memory	Memory			**Occipital cortex**
Executive control	Executive control	Executive control	Thalamus	Thalamus	
			Hippocampus	Hippocampus	
Manual dexterity	Manual dexterity	Manual dexterity	Caudate	Caudate	
Gait and balance	Gait and balance		Putamen	Putamen	
Peripheral neuropathy	Peripheral neuropathy	Peripheral neuropathy		**Pallidum**	
			**Amygdala**		
			**Pons**		
			**Cerebellum**		
**HIV+AUD**			**HIV+AUD**		
Psychomotor speed, memory, executive control, gait and balance			Frontal and temporal cortices, thalamus		
	**HIV+HCV**			**HIV+HCV**	
	Memory, executive control, manual dexterity			Vasculitis	
**MRS: neurometabolite imaging**			**DTI: microstructural imaging**		
**AUD**	**HIV**	**HCV**	**AUD**	**HIV**	**HCV**
Low NAA	Low NAA	Low NAA	Corpus callosum	Corpus callosum	Corpus callosum
**Low Cho**	High Cho	High Cho	(Centrum semiovale)	Corona radiata	Corona radiata
	High mI	High mI	Internal capsules	Internal capsules	
Frontal/cerebellar regions	Frontal/basal ganglia regions	Frontal/basal ganglia/occipital regions	External capsules	External capsules	
			Superior cingulate	Superior cingulate	
			Longitudinal fasciculi		Longitudinal fasciculi
					Cerebellar peduncles
					Fronto-occipital fasciculi
**HIV+AUD**			**HIV+AUD**		
Low NAA			Corpus callosum		
	**HIV+HCV**			**HIV+HCV**	
	High mI			Corona radiata	

## Medical and psychiatric effects of HIV and comorbidities

Age-related medical conditions (e.g., diabetes, hypertension, coronary artery disease, stroke, Alzheimer's disease) are not usually observed in the general population until over age 60: in HIV-infected patients, such conditions may present at middle age or sooner (Guaraldi et al., 2014). HIV-infection is also associated with frailty, the likelihood of which increases with age (Desquilbet et al., 2007). Accelerated aging in HIV may put affected individuals at increased risk for non-HIV-associated cancers (Nasi et al., 2014) and dementias (Verma and Anand, 2014; Sheppard et al., 2015).

HIV infected patients self-report feelings of apathy, lethargy, and depression (Hardy and Vance, 2009; Robertson et al., 2009; Lane et al., 2012; Zayyad and Spudich, 2015). Indeed, aging with HIV may lead to higher rates of psychiatric comorbidities (e.g., major depression, bipolar disorder, anxiety; Valcour et al., 2004; Effros et al., 2008; Leserman, 2008; Havlik et al., 2011). Medical or psychiatric comorbidities in HIV complicate access to care, interfere with self-management, and often necessitate a greater reliance on caregivers.

Because healthy aging results in global increases in immune activation and immune senescence (Schuitemaker et al., 2012), it is thought that a canonically dysregulated immune system (e.g., altered T cell production) can hasten medical or psychiatric disease (Önen and Overton, 2011), thereby contributing to premature or accelerated aging in HIV (Watkins and Treisman, 2012; Zapata and Shaw, 2014).

Medical conditions associated with AUD include liver, lung, and cardiac disease (Simet and Sisson, 2015). AUD-related liver disease has a negative effect on the progression of HIV infection (Petry, 1999; Braithwaite et al., 2007; Soboka et al., 2014; Tran et al., 2014). HIV-infected patients who drink heavily are furthermore at increased risk for cardiovascular disease (Kelso et al., 2015), certain types of cancer (McGinnis et al., 2006), and diabetes (Butt et al., 2009; Wakabayashi, 2014). AUD independently presents with depression and reduced quality of life (Sassoon et al., 2012); alcoholism in HIV likely has an additive effect on depression (Sullivan L. E. et al., 2011), stress, and anxiety (Pence et al., 2008).

HCV liver damage progresses more rapidly in HIV and may accelerate the course and impair the management of HIV (Luetkemeyer et al., 2006; Weber et al., 2006; Chamie et al., 2007; Kim and Chung, 2009; Soriano et al., 2010). In addition, individuals seropositive for HCV have co-occurring insulin resistance beyond what might be predicted by chance (Harrison, 2008). HCV patients frequently report fatigue, lassitude, depression, and poor quality of life (Hilsabeck et al., 2003; Adinolfi et al., 2015). Emerging evidence supports an additive role of HCV and HIV on depression (Ramasubbu et al., 2012), which can negatively impact medical outcomes (Šprah et al., 2017).

## Neuropsychological and motor effects of HIV and comorbidities

HIV-associated neurocognitive disorder (HAND) is ideally assessed using comprehensive neuropsychological batteries and interpreted using demographically appropriate normative data (Antinori et al., 2007). Assessment of HAND allows for grading of functional impairment (Marder et al., 2003; Sacktor et al., 2016), from asymptomatic neurocognitive compromise to HIV-associated dementia (HAD) (Day et al., 1992; Maj et al., 1994; Robertson et al., 2011; Nakazato et al., 2014). The prevalence of HAD on the severe end of the spectrum has declined with ART (Gates and Cysique, 2016). Mild to moderate cognitive deficits in HIV, by contrast, remain an issue (Vivithanaporn et al., 2010; Manji et al., 2013; Underwood et al., 2017b). Despite heterogeneity (Dawes et al., 2008; Vassallo et al., 2015; Joseph et al., 2016), neuropsychological assessments of treatment-stabilized HIV patients often report compromise in domains of attention, psychomotor speed, memory, and executive control (Hinkin et al., 1999; Martin et al., 2003; Becker et al., 2015). Visuospatial abilities are relatively spared (Cysique et al., 2006), but may be sensitive to age-HIV interactions (Foley et al., 2013). Persistent cognitive impairments post-ART have been attributed to a variety of factors (e.g., immunological, genetic, psychosocial) (e.g., Arentoft et al., 2015; Thaler et al., 2015; Hobkirk et al., 2017), including ART, in particular efavirenz (Ciccarelli et al., 2011; Romão et al., 2011; Funes et al., 2014; Ma et al., 2016), advancing age (e.g., Morgan et al., 2011; Brew and Chan, 2014; Jacks et al., 2015; Jiang et al., 2016; Gomez et al., 2017), and comorbidity for substance use (Rosenbloom et al., 2010; Sassoon et al., 2012; Míguez-Burbano et al., 2014) or HCV infection (Devlin et al., 2012).

Motor symptoms described in the treated HIV population include slowing, clumsiness, poor balance, and loss of fine motor control (Fama et al., 2007; Robertson et al., 2007; Sullivan E. V. et al., 2011; Bernard et al., 2013; Wilson et al., 2013; Prakash et al., 2016). Peripheral neuropathy, a persisting and prevalent (15–40%, Newton, 1995; Evans et al., 2008) HIV-associated disturbance in the post-ART era (Geraci and Simpson, 2001; Robertson et al., 2011; Kranick and Nath, 2012; Gabbai et al., 2013), is also associated with older age (Saylor et al., 2017) and ART (Dragovic and Jevtovic, 2003; Venhoff et al., 2010; Birbal et al., 2016; Weldegebreal et al., 2016; Adoukonou et al., 2017; Benevides et al., 2017) and likely contributes to impaired motor control.

Indeed, toxicity of ART goes beyond originally reported side effects of medications. An unexpected relationship between high current CD4 and deterioration of clinical status is an active area of investigation (e.g., Jernigan et al., 2011; Nasi et al., 2017) and a growing concern for the aging HIV population (Manji et al., 2013; Zaffiri et al., 2013). This condition, referred to immune reconstitution inflammatory syndrome (IRIS), applies to HIV patients who experience worsening symptoms as a result of anti-retroviral therapy mediated immune restoration (Venkataramana et al., 2006; Johnson and Nath, 2010). The effects of IRIS on brain structure may not be visible with conventional MRI (Narvid et al., 2016), but may be detectable with quantitative diffusion tensor imaging (DTI) (Zhu et al., 2013), which focuses on the integrity of white matter microstructure.

To account for variability seen in neuropsychological performance in cross-sectional studies (Schretlen et al., 2003), it has been posited that an increase over time (6-month interval) in intra-individual variability (or dispersion) in neurocognitive performance contributes to poorer antiretroviral medication adherence, which in turn can lead to additional neurocognitive impairments, precipitating a deteriorating cycle (Thaler et al., 2015). The Multicenter AIDS Cohort Study (MACS) enrolled a total of 6972 men from sites in Baltimore, Washington, Chicago, Los Angeles, and Pittsburgh at three separate time points: in 1984–1985, 1987–1991, and 2001–2003. Neuropsychological evaluation included measures from multiple domains. A data-driven Mixed Membership Trajectory Model technique was used to investigate potential trajectories of cognitive impairment. The findings suggest three distinct trajectories: “normal aging” was defined as a low probability of mild impairment until age 60; “premature aging” was defined as mild impairment starting at age 45–50 (i.e., “premature aging” relative to “normal aging” was offset to the left by 25+ years); “unhealthy aging” was defined as mild impairment at ages 20–39. Clinically defined AIDS, HCV-infection, depression, and race affected an individual's trajectory classification (Molsberry et al., 2015). Our work comports with the results of the MAPS study showing that cognitive performance slope differences between control and HIV groups can be modeled as premature aging, in that differences between the patients and the controls occur without interactions with aging (Pfefferbaum et al., 2014).

Studies focused on neuropsychological performance in AUD show impairments in memory, psychomotor speed, and executive functioning: problems in visuospatial and emotional regulation domains appear to be unique to AUD (Chanraud et al., 2007; Fama et al., 2009; Oscar-Berman et al., 2014; Wilcox et al., 2014; Le Berre et al., 2017). Motor effects of AUD include compromise of upper limb motor abilities, and gait and balance (Sullivan et al., 2000b,c, 2002; Vassar and Rose, 2014). Peripheral neuropathy reported in AUD has been related to nutritional deficiencies (Chopra and Tiwari, 2012; Noble and Weimer, 2014).

**Figure 2:**
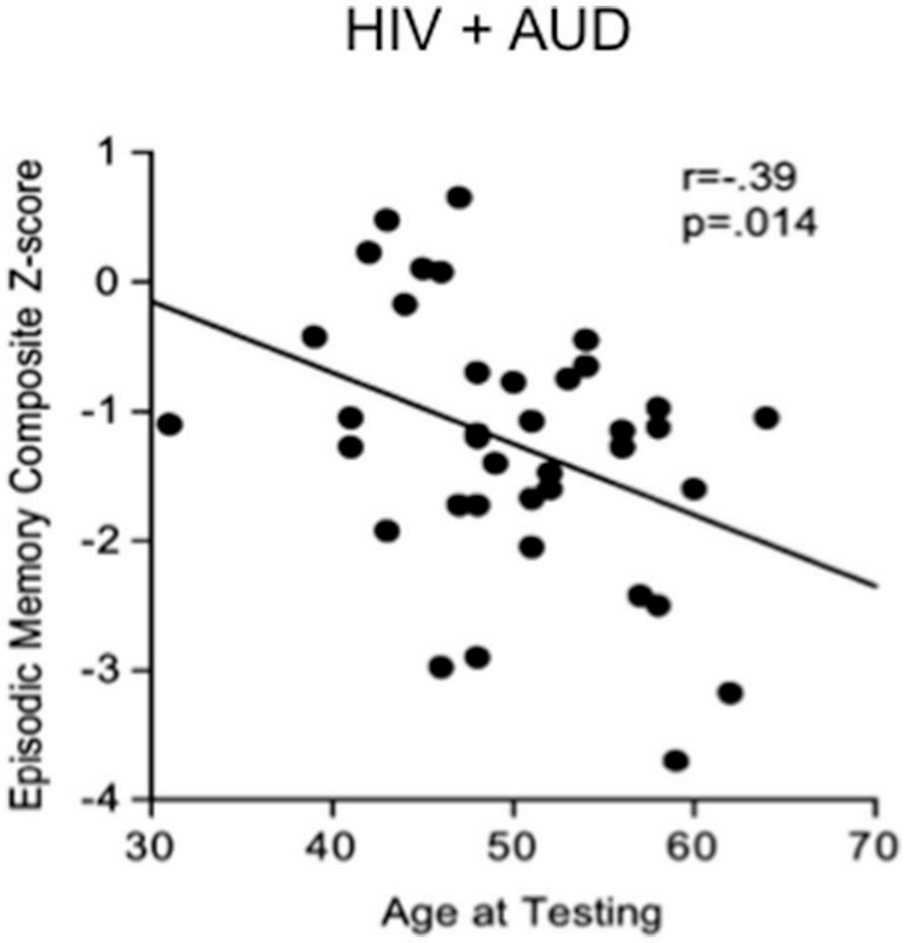
Scatterplot depicting significant relationship between age and episodic memory in HIV+AUD comorbidity**:** poorer scores in older age, despite age-corrected Z-scores. Reprinted from Fama et al. (2016), with permission from John Wiley and Sons.

Substance abuse can independently contribute to neuropsychological impairments in HIV (e.g., Gomez et al., 2017). In a recent study, 52% of HIV positive patients showed cognitive deficits, often related to high alcohol consumption (McNamara et al., 2017). In studies aimed at discerning the independent effects of HIV and AUD (e.g., Fama et al., 2012), impairments in planning and free recall of visuospatial material marked AUD, whereas impairments in psychomotor speed, sequencing, narrative free recall, and pattern recognition marked HIV. Our work demonstrates that tests of executive function, episodic memory, and processing efficiency (expressed as age- and education-corrected composite Z-scores) show a graded effect, with HIV+AUD performing worse than controls on executive function and episodic memory and worse than AUD alone or HIV alone on episodic memory (Fama et al., 2016): in HIV+AUD, age was a unique predictor of poor episodic memory (Figure 2). Our work comports with the literature that comorbidity for HIV+AUD results in compounding effects (Rothlind et al., 2005; Fama et al., 2014) on declarative memory (Fama et al., 2009, 2016), remote memory (Fama et al., 2011), selective attention and conflict processing (Schulte et al., 2005), psychomotor speed (Sassoon et al., 2007), gait and balance (Fama et al., 2007), and quality of life (Rosenbloom et al., 2007).

HCV-infected individuals experience cognitive decline even in the absence of cirrhosis-associated hepatic encephalopathy or other indices of liver damage (Karaivazoglou et al., 2007). Some groups have argued that cognitive deficits in HCV are due to interferon treatment (Asnis and Migdal, 2005; Capuron et al., 2005; Reichenberg et al., 2005), but cognitive deficits persist despite successful antiviral (interferon) therapy (Thein H. H. et al., 2007; Weissenborn et al., 2009; Cattie et al., 2014; Kuhn et al., 2017). Although the literature is heterogeneous and characterized by cross-sectional rather than longitudinal assessments of relatively small and select cohorts, neurocognitive deficits reported in HCV include compromised attention, memory, and psychomotor speed (Forton et al., 2002; Hilsabeck et al., 2002; Capuron et al., 2005; Iriana et al., 2017) with fewer reports of deficits in executive functioning (Córdoba et al., 2003; Weissenborn et al., 2004), fine-motor coordination (Vigil et al., 2008), and presence of peripheral neuropathy (Adinolfi et al., 2015; Mathew et al., 2016).

Studies reporting on the combined effects of HIV and HCV on neuropsychological performance suggest that the two viruses result in similar neurocognitive consequences (cf., Parsons et al., 2006; Thein H. et al., 2007; Martin-Thormeyer and Paul, 2009; Martin et al., 2015; Molsberry et al., 2015) with comorbidity associated with greater neurocognitive impairment than in either infection alone (Hilsabeck et al., 2003; von Giesen et al., 2004; Cherner et al., 2005; Letendre et al., 2005; Richardson et al., 2005; Sun et al., 2013; Caldwell et al., 2014; but see: Perry et al., 2005; Soogoor et al., 2006; Clifford et al., 2015), particularly on measures of memory (Hilsabeck et al., 2005; Hinkin et al., 2008), executive functioning (Ryan et al., 2004), and motor dexterity (Cherner et al., 2005).

In summary, available evidence suggests that neurocognitive performance in ART-treated HIV individuals shows premature aging. HIV, AUD, and HCV can independently impair neuropsychological functioning and appear to have additive effects on some domains of cognition, which in practice can have significant effects on key outcomes such as employment status (van Gorp et al., 1999; Heaton et al., 2004), medication adherence (Hinkin et al., 2004), and driving safety (Marcotte et al., 2006).

## *In vivo* neuroimaging of HIV and comorbidities

### Macrostructural magnetic resonance imaging (MRI)

In the ART era, clinical MRI scanning reveals relatively few gross intracranial abnormalities in HIV, particularly when neurological signs are absent (Nishijima et al., 2014). Although severe brain atrophy is uncommon in HIV stabilized by treatment, brain volume deficits can be detected with quantitative methods in select regions of the cortex, basal ganglia, and cerebellum (Aylward et al., 1993; Di Sclafani et al., 1997; Stout et al., 1998; Tagliati et al., 1998; Ragin et al., 2012; Kallianpur et al., 2013; Underwood et al., 2017a). Cortical areas with gray matter volume deficits in HIV with viral suppression, relative to healthy controls, include frontal, cingulate, sensorimotor, and parietal regions (Heaps et al., 2012; Li et al., 2014; Pfefferbaum et al., 2014; Clark et al., 2015; Janssen et al., 2015; Wang et al., 2016). Those without complete viral suppression exhibit greater volume deficits than virally-suppressed individuals (Cardenas et al., 2009; Kallianpur et al., 2013; Hines et al., 2016). The imaging literature typically reports the effects of HIV on gray matter volume [see the following for exceptions] (Corrêa et al., 2016a; du Plessis et al., 2016; Castillo et al., 2017). In studies that assessed cortical thickness rather than cortical volume, HIV effects can be evident in areas such as the insula and temporal cortices (Kallianpur et al., 2012; Sanford et al., 2017).

Subcortical regions with significantly smaller volumes, particularly in older HIV subjects relative to healthy controls, include thalamus, hippocampus, caudate, putamen, and pallidum (Dewey et al., 2010; Li et al., 2013; Wade et al., 2015; du Plessis et al., 2016; Wright et al., 2016; Sanford et al., 2017). Brain tissue abnormalities have been reported to correlate with nadir CD4 cell counts (Thompson et al., 2005; Jernigan et al., 2011; Kallianpur et al., 2012; Hua et al., 2013). However, HIV individuals with an active life style (energy use above resting expenditure) were found to have a larger putamen (Ortega et al., 2015), and longitudinal study reveals that increasing CD4 counts (notwithstanding IRIS) are associated with increases in subcortical gray matter volumes (Fennema-Notestine et al., 2013) and slower tissue volume declines (Pfefferbaum et al., 2014).

As described for compromised neuropsychological performance in HIV, brain volume deficits in the ART era may be associated with more traditional risk factors (e.g., age, education, diabetes) than with HIV-related variables (Bonnet et al., 2013; Lake et al., 2017; but see Ragin et al., 2011; Kallianpur et al., 2016). Although HIV and aging appear to contribute independently to heighten brain structural vulnerability (Ances et al., 2012), HIV may accelerate brain aging (Cysique et al., 2013; Cole et al., 2017). Consequently, despite persistent control of plasma viremia, older HIV infected patients demonstrate more rapid progressive brain compromise when compared to healthy aging (Clifford et al., 2017).

The few published longitudinal volumetric MRI studies have been conducted over relatively brief intervals, typically 1–2 years. An initial study found faster rate of cortical volume decline in mild (CDC stage A) and severe (CDC stage C) stages of HIV infection relative to changes observed in infection-free controls and faster rates of white matter volume decline in the HIV-infected subgroup with stage C than stage A severity level. Further, decline in caudate nucleus volume and increase in ventricular volume were greater in the HIV-infected group that progressed from a less severe to a more severe CDC stage across MRI sessions, and these changes in brain volumes correlated with decline in CD4 cell count (Stout et al., 1998). A 2-year longitudinal study indicated widespread white matter volume loss and posterior gray matter loss (parietal, occipital, and cerebellar) in virally-suppressed HIV individuals, depending on analysis approach; those without complete viral suppression exhibited accelerated volume loss in gray and white matter compared with declines measured in controls (Cardenas et al., 2009). Examination of HIV infected individuals before and about 6 months after starting ART revealed improvement in neuropsychological test performance but no appreciable change in regional brain volumes (Ances et al., 2012). In this relatively small study, older age and HIV infection were independently related to smaller volumes of the caudate, with evidence for premature aging of the caudate in HIV-infected participants, while volumes of the amygdala and corpus callosum were sensitive to HIV but not aging.

**Figure 3:**
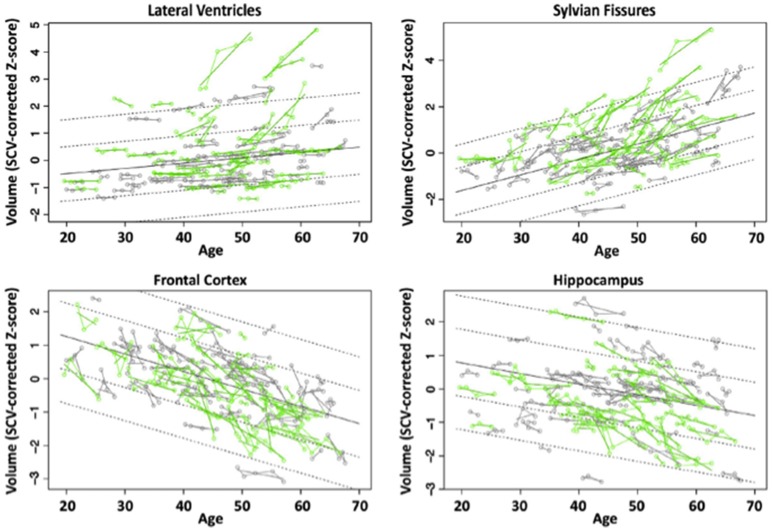
Plots of individual supratentorial cranial volume (SCV)-corrected Z-scores by age for each control (gray) and each HIV-infected participant (green) for the lateral ventricles, Sylvian fissures, frontal cortex, and hippocampus. Each participant's values are connected over time and the age-centered slope of each participant is overlaid on his or her longitudinal data points. The long solid black regression line is the expected volume by age regression based on the controls; dotted lines are ±1 and 2 standard deviations. Reprinted from Pfefferbaum et al. (2014) with permission from Elsevier.

We evaluated brains of 51 HIV and 65 controls from 351 longitudinal MRI scans and concurrent neuropsychological evaluation collected 2 or more times over 6 months to 8 years (Pfefferbaum et al., 2014). Although HIV individuals were in good general health and free of clinically detectable dementia, significant volume effects, where HIV-infected participants had greater volumes in CSF regions and smaller volumes in tissue regions than controls, were found in the Sylvian fissures, cingulum, insula, thalamus, and hippocampus. Significant slope effects, where the HIV-infected group showed greater change per year over the years of observation than the control group, were detected in the lateral ventricles, insula, and hippocampus. Greater acceleration in slope with advancing age in the HIV-infected individuals was found for frontal, temporal, and parietal cortices and thalamus (Figure 3). In this study, the most consistent and robust predictors of brain volume trajectories were CD4 count and duration of HIV infection (Pfefferbaum et al., 2014).

Effects of HIV and comorbid substance abuse on brain structure can depend on the substance and quantity consumed [e.g.,] (Durazzo et al., 2007; Thames et al., 2017). In AUD, volume deficits are evident in brain regions including frontal cortex (Pfefferbaum et al., 1997; Cardenas et al., 2005, 2007), cerebellum (i.e., hemispheres; Sullivan et al., 2000a,c; De Bellis et al., 2005; Chanraud et al., 2007, 2009a; Boutte et al., 2012), pons (Pfefferbaum et al., 2002a; Sullivan, 2003; Chanraud et al., 2009b), mammillary bodies (Shear et al., 1996; Sullivan et al., 1999), hippocampus, thalamus (Sullivan, 2003; De Bellis et al., 2005; Chanraud et al., 2007; Pitel et al., 2012; van Holst et al., 2012), caudate (Boutte et al., 2012), putamen (Jernigan et al., 1991), amygdala (Fein et al., 2006), and nucleus accumbens (Sullivan et al., 2005). Those with both HIV infection and alcoholism show ventricular enlargement greater than in either condition alone (Rosenbloom et al., 2010). Quantitative analysis of MRI brain structural data from cross-sectional study of 4-groups (controls, AUD, HIV, HIV+AUD) revealed regional volume deficits in all 3 patient groups: HIV alone had relatively few deficits, except in thalamus (Pfefferbaum et al., 2012), as has recently been replicated (Janssen et al., 2015); HIV+AUD showed moderate to severe abnormalities affecting multiple brain regions (e.g., frontal and temporal cortices, thalamus, corpus callosum, Sylvian fissure, 3rd ventricle); and HIV+AUD with an AIDS diagnosis had the most serious untoward effects on brain structure (Pfefferbaum et al., 2012).

In non-cirrhotic HCV patients relative to controls, a recent study suggests that cortical thickness is reduced in frontal and occipital cortices (Hjerrild et al., 2016; also see Iwasa et al., 2012). HIV + HCV co-infection has been associated with increased incidence of neurovascular disease (Jernigan et al., 2011; Ojaimi et al., 2014; but see Ramos-Casals et al., 2007) and compromised brain perfusion (Bladowska et al., 2014), but the effects of HIV and HCV co-infection on brain macrostructural integrity is an area for further investigation. In summary, structural imaging suggests that HIV infection may lead to accelerated aging of the brain, which is compounded by AUD comorbidity, particularly in subcortical regions such as the thalamus. Additional work is required to determine whether non-cirrhotic HCV is associated with regional brain volume deficits and whether HIV+HCV co-infection has additive effects on reducing regional brain volumes.

### White matter hyperintensities

White matter damage can be measured by examining white matter hyperintensities (WMH) on fluid attenuated inversion recovery (FLAIR) images from MRI. WMH may reflect vascular or inflammatory brain changes (Maniega et al., 2015; Shoamanesh et al., 2015). The prevalence of cerebrovascular events in HIV remains higher, in relatively younger patients, despite treatment, than in the general population (Haddow et al., 2014; Arentzen et al., 2015). The frequency of cerebrovascular disease increases with age (Kendall et al., 2014) and HIV individuals with cerebrovascular disease are more likely to have cognitive deficits (Foley et al., 2008; Nakamoto et al., 2012).

WMH are a frequent finding on brain MRI of elderly subjects (over aged 60) and associated with hypertension (e.g., Rostrup et al., 2012; Peng et al., 2014). A number of studies report a greater prevalence of WMH in HIV relative to healthy controls (Foley et al., 2008; Su et al., 2016), specifically affecting frontal lobes (McMurtray et al., 2008). While one study reports that with older age, patients with HIV have a greater number of WMH relative to age-matched healthy controls related to a history of AIDS, current CD4, and active HCV infection (Seider et al., 2016), another found a similar number of WMH volumes in HIV and controls (Watson et al., 2017), explained by hypertension (Su et al., 2016; Watson et al., 2017).

There is little evidence that alcohol consumption increases WMH load (e.g., Anstey et al., 2006). In non-cirrhotic HCV patients relative to controls, imaging provides evidence for an increased incidence of WMH representing cerebral vasculitis (Heckmann et al., 1999; Casato et al., 2005; Bezerra et al., 2011). Indeed, in HIV, the presence of HCV was the strongest predictor of WMH (Robinson-Papp et al., 2017).

### Structure/function relationships

A primary goal of evaluating structure/function relationships in HIV is to advance understanding of the neural substrates of HIV-associated motor and cognitive compromise. Significant, but non-specific correlations have been reported between the severity of global brain atrophy and general cognitive impairment in HIV (Becker et al., 2012; Steinbrink et al., 2013; but see Heaps et al., 2015). By contrast, cortical thinning of the retrosplenial cortex has been proposed as a selective contributor to general cognitive impairment in HIV (Shin et al., 2017). A number of studies report deficits in regional brain volumes associated with poor cognitive performance in HIV: the caudate with psychomotor performance (Kieburtz et al., 1996; Paul et al., 2002; Kallianpur et al., 2016); the anterior cingulate with emotion processing (Clark et al., 2015); the prefrontal cortex with verbal learning and memory (Rubin et al., 2016). The assortment of brain regions implicated likely reflects heterogeneity in disease course. Indeed, post-ART, global, cortical-driven pathogenesis rather than subcortical dysfunction is a more likely contributor the varying HIV clinical manifestations (Foley et al., 2008). Cognitive heterogeneity post-ART thus requires further evaluation of select brain structure/function relationships, particularly in stably-treated, aging HIV cohorts, with comorbid risk factors.

In HIV-infected alcoholics, smaller thalamic volumes were associated with poorer performance on tests of explicit (immediate and delayed) and implicit (visuomotor procedural) memory (Fama et al., 2014), again indicating the thalamus as a structure that is particularly susceptible to HIV and the compounding effects of AUD. The potential for segmentation of thalamic subregions (Behrens et al., 2003; Deoni et al., 2007; Zhang et al., 2010; Deistung et al., 2013; Kim et al., 2013; Barron et al., 2014) holds promise for a more refined understanding of brain structure/function relationships and affected neural circuitry (Fama et al., 2016) in HIV.

### Magnetic resonance spectroscopy (MRS)

MRS is a modality used to quantify brain metabolites, typically N-acetyl aspartate (NAA), choline-containing compounds (Cho), and total creatine (tCr). NAA is an indicator of neuronal integrity, with decreases suggesting neuronal dysfunction (e.g., Zahr et al., 2010, 2013). The signal from Cho, including contributions from free choline, glycerophosphorylcholine, and phosphorylcholine (Miller, 1991), is a marker for cell membrane synthesis and turnover. The signal from tCr, with contributions from creatine and phosphocreatine, represents the high-energy biochemical reserves of neurons and glia (Inglese et al., 2003). Less frequently reported, as their quantification is more challenging, are levels of myo-Inositol (mI) and glutamate (Glu). Because mI, an osmolyte, is primarily present in glial cells (Brand et al., 1993), it is considered a glial marker. Glu is a ubiquitous molecule used in cellular metabolism and is the principal excitatory neurotransmitter (Thangnipon et al., 1983; Fonnum, 1984).

MRS studies of HIV patients commonly report that neuronal injury (dysfunction or loss) is associated with low levels of NAA and changes (both increases and decreases) in Glu levels (often quantified from the combined resonance of glutamate + glutamine and referred to as Glx) in regions including frontal cortex and basal ganglia (López-Villegas et al., 1997; Chang et al., 1999, 2013; Paul et al., 2008; Hua et al., 2013; Harezlak et al., 2014; Bairwa et al., 2016); longitudinal: (Lentz et al., 2011; Sailasuta et al., 2012; Gongvatana et al., 2013; Young et al., 2014; Scott et al., 2016; Rahimy et al., 2017). Similar findings of abnormally low NAA (McAndrews et al., 2005) are also reported in HCV in regions such as the occipital cortex (Weissenborn et al., 2004); but see (Bokemeyer et al., 2011).

During acute/early infection and at two follow-up time points (2 and 6 months), greater numbers of activated (CD16+) monocytes were associated with lower NAA and higher Cho levels in frontal cortex (Lentz et al., 2011). Similarly, above control levels of Cho were identified in basal ganglia in acute HIV; these resolved to control levels at 6 month following initiation of ART (Sailasuta et al., 2012). Similar findings (longitudinal increases in Cho) were reported in frontal white matter and parietal gray matter prior to ART initiation, with resolution following ART (Young et al., 2014). By contrast, a study in chronic HIV with longer intervals between MRS showed that despite stable ART and virological suppression, and in both asymptomatic and cognitively impaired subgroups, HIV-infected subjects showed significant annual decreases in brain metabolites (including NAA, Cho, tCr, and Glx) in midfrontal cortex, frontal white matter, and basal ganglia (Gongvatana et al., 2013).

Most MRS studies show lower levels of NAA in recently sober alcoholics relative to healthy subjects in several brain regions including frontal areas (Fein et al., 1994; Jagannathan et al., 1996; Seitz et al., 1999; Bendszus et al., 2001; Schweinsburg et al., 2003; Durazzo et al., 2004, 2010; Meyerhoff et al., 2004) and cerebellum (Jagannathan et al., 1996; Seitz et al., 1999; Bendszus et al., 2001; Parks et al., 2002; Durazzo et al., 2010). Neuronal compromise (reduced NAA) appears to be compounded in HIV+AUD (Pfefferbaum et al., 2005). Below control levels of Cho in AUD patients shortly following detoxification are also reported in frontal (Fein et al., 1994; Durazzo et al., 2004; Ende et al., 2005) and cerebellar (Seitz et al., 1999; Bendszus et al., 2001; Parks et al., 2002; Ende et al., 2005; Pfefferbaum et al., 2005; but see Modi et al., 2011; Hermann et al., 2012) regions.

Neuroinflammation in either HIV or HCV has been associated with elevated levels of mI, Cho, and tCr in frontal and basal ganglia regions (Chong et al., 1993; English et al., 1997; Forton et al., 2001, 2002, 2008; Chang et al., 2002, 2013, 2014; Fuller et al., 2004; Weissenborn et al., 2004; McAndrews et al., 2005; Grover et al., 2012; Bladowska et al., 2013). MRS studies of HIV + HCV suggest that co-infection might be associated with higher mI (Garvey et al., 2012) and less variability and more reliability in reported metabolite changes (Vigneswaran et al., 2015).

**Figure 4:**
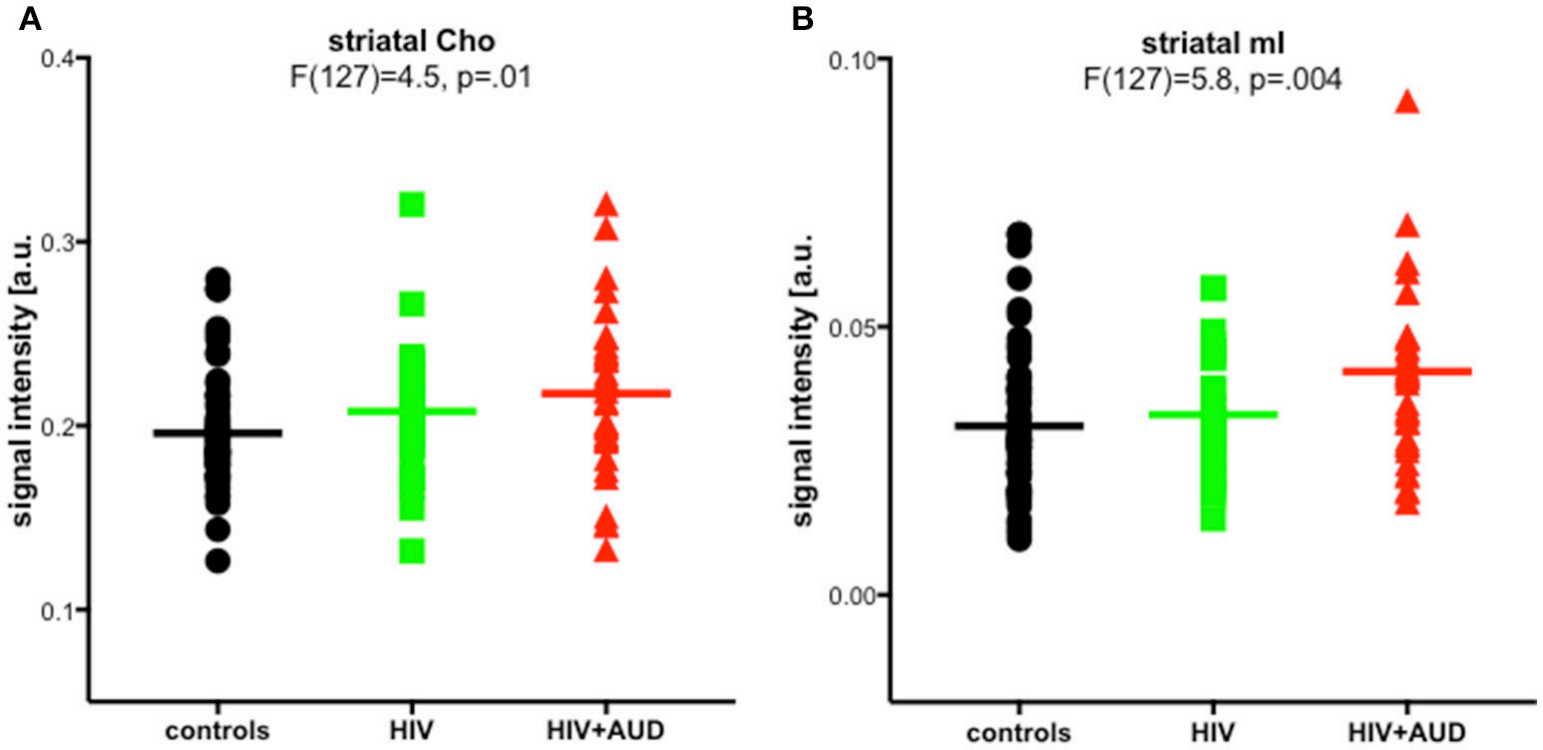
Striatal choline-containing compounds (Cho) and myo-Inositol (mI) levels across 3 groups (controls, HIV, HIV+AUD). Reprinted from Zahr et al. (2014) with permission from John Wiley and Sons.

In a previously published work, we challenged the specificity of Cho and mI as markers of neuroinflammation. Significant group effects were evident for striatal Cho and striatal mI, higher in HIV+AUD than in controls (Figure 4). Correlations evaluated in HIV groups only (i.e., HIV, HIV+AUD) demonstrated that having HCV or an AIDS-defining event was associated with higher Cho; lower Cho levels, however, were associated with low thiamine levels and with ART. Higher levels of mI were related to greater lifetime alcohol consumed, whereas ART was associated with lower mI levels (Zahr et al., 2014). These results demonstrate that competing mechanisms can influence Cho and mI levels, and that elevations in these metabolites cannot necessarily be interpreted as reflecting a single underlying mechanism such as neuroinflammation.

### Microstructural diffusion tensor imaging (DTI)

Examination of brain microstructural integrity using DTI has detected subtle HIV-related differences from controls [e.g., low fractional anisotropy (FA) and high mean diffusivity (MD)] in markers of myelin (radial or transverse diffusivity) and axonal (axial or longitudinal) integrity, even in normal-appearing white matter, notably in corpus callosum and frontal lobe white matter (e.g., Pfefferbaum et al., 2007, 2009a; Chen et al., 2009; Hoare et al., 2011; Towgood et al., 2011; Du et al., 2012). Variable results from DTI studies may be due, at least in part, to timing of evaluation relative to treatment (i.e., treatment naïve, currently un-medicated, chronically medicated, or older HIV infected individuals). For example, in early, treatment naïve HIV infection, white matter impairment (Tang et al., 2017) correlated with days since infection (Wright et al., 2015). In those on ART, a number of fiber tracts, including those of the corpus callosum and corona radiate are often reported as compromised (Leite et al., 2013; Xuan et al., 2013; Su et al., 2016; Wang et al., 2016). Effects on DTI metrics may also depend on presence of neurological complications (Corrêa et al., 2015), with symptomatic individuals showing effects extending to frontal areas (Zhu et al., 2013). Chronic relative to initial infection often shows more substantial differences in DTI metrics related to biomarkers of infection (e.g., viral load and immune compromise), disease duration, and ART duration (Wright et al., 2015; Cordero et al., 2017; Strain et al., 2017), which complicates attempts to distinguish effects of age, as age is often correlated with the duration of infection and ART.

Most DTI studies report independent effects of age and HIV on DTI metrics, but no evidence for an interaction (Gongvatana et al., 2011; Towgood et al., 2011), even in subjects over the age of 60 (Nir et al., 2014). Instead, for example, longer HIV duration may interact with the presence of the apolipoprotein E4 allele (which increases the risk for Alzheimer's disease) (Jahanshad et al., 2012; Wendelken et al., 2016) or impaired glucose metabolism (Nakamoto et al., 2012) to compromise the brain in older HIV-infected individuals. A single study reported significant age by HIV interactions for decreased FA in the posterior limbs of the internal capsules, cerebral peduncles, and anterior corona radiata in HIV+ relative to seronegative control participants (Seider et al., 2016); HIV duration as measured by time since diagnosis was not a significant predictor of white matter damage in the described cohort suggesting that the reported interaction truly reflected the effects of aging. Support for an interactive effect of aging and HIV on DTI metrics comes from a longitudinal DTI study suggesting greater than normal age-related changes on the genu of HIV patients at 1 year follow up (Chang et al., 2008). A more recent longitudinal study, with an approximate 2-year follow-up interval, did not show differences in metrics between the first and second evaluation (Corrêa et al., 2016b), possibly because viremia was better controlled in the later study.

Although widespread abnormalities in white matter microstructure correlate with general cognitive compromise in HIV (Nir et al., 2014; Strain et al., 2017; Underwood et al., 2017a; Watson et al., 2017), more specific microstructure/function relationships have also been reported. For example, planning deficits correlated with low FA in anterior thalamic radiations, inferior fronto-occiptal fasciculi, superior longitudinal fascicule, corpus callosum genu, and uncinate fascicule (Corrêa et al., 2015); motor impairments correlated with low FA in various motor tracts (Bernard et al., 2013); self-reported signs of peripheral neuropathy correlated with abnormally high callosal diffusivity (Pfefferbaum et al., 2009a).

DTI has revealed microstructural damage related to alcoholism in cerebral areas that appear intact in structural MRI analyses (e.g., Pfefferbaum and Sullivan, 2002; Sullivan et al., 2003; Pfefferbaum et al., 2006). Quantitative fiber tracking has demonstrated in alcoholics compared with controls greater FA deficits in anterior than in posterior fibers of supratentorial and infratentorial white matter bundles as well as low FA in tracts of the corpus callosum, centrum semiovale, internal and external capsules, fornix, superior cingulate, longitudinal fasciculi (Pfefferbaum et al., 2000, 2002b, 2009b; Pfefferbaum and Sullivan, 2005; Müller-Oehring et al., 2009; Trivedi et al., 2013; Fortier et al., 2014).

Quantitative analysis DTI data from cross-sectional study of 4-groups (controls, AUD, HIV, HIV+AUD) revealed in all patient groups relative to controls lower integrity of callosal regions (Pfefferbaum et al., 2007) and uncinate fasciculus (Schulte et al., 2012): degradation of callosal microstructure showed evidence for compounded AUD+HIV effects (Pfefferbaum et al., 2007).

In HCV, FA has been reported as low in fiber tracks including the corpus callosum, middle cerebellar peduncles (Bladowska et al., 2013), external capsules, fronto-occipital fasiculi (Bladowska et al., 2013; Thames et al., 2015), longitudinal fasciculi (Bladowska et al., 2013; Kuhn et al., 2017), and corona radiata (Kuhn et al., 2017). Studies of HIV+HCV co-infection show greater brain-wide diffusivity with voxel-based analysis (Stebbins et al., 2007) and higher diffusivity and lower FA by region-of-interest analysis (Gongvatana et al., 2011). A study evaluating co-infection on corpus callosum microstructure reported no additive effects (Heaps-Woodruff et al., 2016), whereas another study using TBSS noted compromise of the corona radiata in HIV + HCV co-infection (Seider et al., 2016).

## Summary and conclusions

Getting old with HIV appears to cause premature aging with respect to medical conditions, psychiatric comorbidities, and neurocognitive performance. Structural MRI findings suggest accelerated aging of select brain gray matter volumes, but equivocal evidence for an interactive increase in WMH burden in older HIV-infected individuals. Current DTI studies are similarly conflicting as to whether older age and HIV have interactive effects on white matter integrity. The literature remains sparse with respect to longitudinal studies, which will help distinguish between healthy, premature, and accelerated aging with HIV.

ART has largely controlled the HIV epidemic, but fundamental questions regarding the precise cause of neurocognitive dysfunction in HIV remain. In the post-ART era, persistent issues related to an aging HIV population include effects of common comorbid conditions, such as AUD and HCV infection. Neuroimaging points to the sensitivity of the thalamus to HIV infection. High-resolution imaging and segmentation of thalamic substructures may provide a more refined understanding of the substrates underlying cognitive decline in HIV. DTI has been underutilized in studying the HIV brain and thus also holds promise for clarifying the brain regions involved in HIV-associated cognitive and motor impairments and in explicating mechanisms that may contribute to dysfunction with age. Free water imaging, a DTI analysis method that improves the specificity and sensitivity of DTI by accounting for extracellular water (Pasternak et al., 2009, 2012; Metzler-Baddeley et al., 2012), may permit a better understanding of neuroinflammatory processes in HIV (Strain et al., 2017) and aging. A better understanding of the aging HIV brain will help in the development of integrated healthcare approaches for these complicated patients.

## Author contributions

NMZ envisioned and wrote this review manuscript and is accountable for all aspects of the work.

### Conflict of interest statement

The author declares that the research was conducted in the absence of any commercial or financial relationships that could be construed as a potential conflict of interest.
